# Knowledge about hereditary nonpolyposis colorectal cancer; mutation carriers and physicians at equal levels

**DOI:** 10.1186/1471-2350-10-30

**Published:** 2009-03-26

**Authors:** Katarina Domanska, Christina Carlsson, Pär-Ola Bendahl, Mef Nilbert

**Affiliations:** 1 Department of Oncology, Institute of Clinical Sciences, Lund University, Sweden; 2 Clinical Research Centre, Hvidovre Hospital, Copenhagen University, Hvidovre, Denmark

## Abstract

**Background:**

Identification and adequate management of individuals at risk for hereditary nonpolyposis colorectal cancer (HNPCC) is crucial since surveillance programmes reduce morbidity and mortality. We investigated knowledge about key features of HNPCC in at risk individuals and physicians in surgery, gynecology and oncology.

**Methods:**

Data were collected using a questionnaire which was answered by 67 mutation carriers and 102 physicians from the southern Swedish health care region. The statements were related to colorectal cancer, heredity and surveillance and the physicians were also asked questions about cancer risks and surveillance strategies.

**Results:**

Both groups answered questions on colorectal cancer risk, surveillance and genetic testing well, whereas answers about inheritance and risks for HNPCC associated cancer were less accurate. Only half of the family members and one third of the physicians correctly estimated the risk to inherit an HNPCC predisposing mutation. Among family members, young age (<57 years), female sex and recent genetic counseling significantly correlated with better results. Physicians generally underestimated the risk of HNPCC associated cancers and three out of four suggested a later starting age for surveillance than recommended.

**Conclusion:**

The finding of similar levels of knowledge about key features of HNPCC in at risk individuals and physicians reflect the challenge physicians face in keeping up to date on hereditary cancer and may have implications for the clinical management and professional relations with HNPCC family members.

## Background

Clinicians are increasingly expected to be familiar with hereditary cancer, including diagnostic criteria, the genetic testing process and recommendations for surveillance and surgery. Though physicians cannot be expected to have detailed knowledge of causative genes and testing platforms, it remains their responsibility to identify at risk individuals and recommend appropriate surveillance. Strategies here for have been adopted by several medical societies and are of particular relevance for physicians in primary health care, surgery, gynecology and oncology [1-6]. At the same time, members in families with hereditary cancer are increasingly well informed through various educational programmes, printed information, internet sites and a growing number of patient associations. Knowledge about hereditary cancer is central and has been demonstrated to correlate with participation in surveillance programmes [1,7,8].

Hereditary nonpolyposis colorectal cancer (HNPCC) is the most common form of hereditary colon cancer with an estimated prevalence of 1/2000 [9]. Colorectal cancer predominates with a life time risk of 60–90%, but female mutation carriers are also at increased risk of gynecological cancer, with a 40–60% risk of endometrial cancer and a 5–15% risk of ovarian cancer [3,10-13]. Various rare tumor types, e.g. cancer in the upper urothelial tract, the small intestine, gastric cancer, brain tumor and skin tumors have also been linked to HNPCC, but the life time risks for these tumors are <5% [10,14-18].

Several features are indicative of HNPCC, e.g. development of multiple HNPCC associated cancers in a family, young age at diagnosis, synchronous and metachronous tumors and certain morphological characteristics. In order to facilitate diagnosis and classification of HNPCC, various guidelines have been developed. The most widely used are the Bethesda guidelines aimed at identifying tumors that should undergo further evaluation for HNPCC, and the Amsterdam criteria aimed at uniform classification of HNPCC families [19-21]. Our study focused on individuals with disease predisposing mismatch repair (MMR) gene mutations, also referred to as Lynch syndrome. International criteria for surveillance of HNPCC families with MMR gene mutations have been published and adopted by the International Society for Hereditary Gastrointestinal Tumors [5,6]. These guidelines recommend colonoscopy biannually from age 20–25 and gynecological examinations with transvaginal ultrasound and endometrial biopsies annually from age 30–35. Whereas colonoscopy surveillance in this high risk population has been proven cost effective, the value of the gynecological surveillance remains unclear [22,23]. Indeed, the high risks of endometrial and ovarian cancer may affirm prophylactic hysterectomy with concomitant oophorectomy after child bearing age [6,24]. Also, the value of gastric cancer and upper tract urothelial cancer surveillance remains unclear, but intervention is recommended from 30–35 years, in families were these tumor types have occurred [6,25].

At risk individuals in HNPCC families are dependent on health care for diagnosis and surveillance, but several studies have suggested that these individuals perceive a lack of knowledge from health care personnel [1,26-28]. We therefore distributed a questionnaire containing key statements related to knowledge of cancer risks and surveillance strategies to mutation carriers in HNPCC families and to physicians in surgery, gynecology and oncology, who are likely to diagnose, treat and survey these families.

## Methods

### Questionnaire

The questionnaire contained 11 statements related to colorectal cancer in general (questions 1 and 3), HNPCC cancer risk (questions 2, 4 and 5), surveillance (questions 6 and 7), heredity (questions 8 and 11) and genetic testing (questions 9 and 10); the statements were to be marked "true" or "false" (Additional file 1). Demographic data were also collected. The questionnaire distributed to the physicians also contained questions related to risk levels (<5%, 5–20%, 20–40%, 40–60%, 60–80% and >80%) for colorectal cancer, endometrial cancer, ovarian cancer, gastric cancer and upper tract urothelial cancer and initiation age (20–25, 25–35, 35–45 or >45 years) for surveillance programmes of HNPCC associated cancers.

### Participants

The questionnaire and a letter of invitation to the study were mailed to 88 individuals with HNPCC predisposing MMR gene mutations. All individuals had undergone genetic counseling and testing at Lund University Hospital between 1996 and 2008. They had been recommended surveillance programmes at their local hospitals and those diagnosed after 2003 also received a HNPCC information booklet. Data were obtained from 67 (76%) individuals without differences in age, sex or time since genetic counseling for responders and non responders. Among the responders the median age was 49 (22–81) years with 38 (57%) being female and 19 (28%) representing index individuals in their families. Educational level was elementary school in 27%, high school in 31%, university in 27% and other in 15% of the at risk individuals. The median time since genetic counseling was 4 (0–12) years and 28 (42%) had a personal history of cancer.

In total, 103 physicians from 6 departments (2 university hospitals and 3 regional hospitals) in the southern Sweden health care region were invited to participate during staff meetings. After filling out the questionnaire, the authors presented updated guidelines for identification, genetic testing and surveillance of HNPCC. Responses were obtained from 102 (99%) physicians, with a median age of 47 (22–64) years and 48 (47%) being female. Specialization was surgery in 40%, gynecology in 29% and oncology in 30% of the informants. The physicians reported median 12 (0–39) years in clinical practise. 72% worked at regional hospitals and 28% were employed at a university hospital.

Ethical approval for the study was granted from the Lund University ethics committee (346/2007).

### Statistical analysis

STATA (StataCorp. 2005. *Stata Statistical Software: Release 9*. College Station, TX: StataCorp LP) was used for the statistical analyses. For each of the eleven questions, the Chi-squared test was used for comparison of the fraction of correct answers among mutation carriers and physicians, whereas Kruskal-Wallis test was applied to compare the distribution of correct answers (range 0–11) in two or more groups. Linear regression was used for multivariate analysis. Unanswered questions were interpreted as missing values. All tests were two-tailed and p-values < 0.05 were considered significant.

## Results

### Key statements among at risk individuals

The median number of correctly answered questions was 9 (3–11) of 11 (Additional file 1). Questions about colorectal cancer in general, HNPCC associated cancer risk and genetic testing had a high number of correct answers. Questions 3 (*individuals not carrying a HNPCC mutation will never develop colorectal cancer*) and 7 (*individuals that carry HNPCC mutations need regular colonoscopies*) had the highest frequencies, 99% and 96%, of correct answers. Knowledge about risks for other cancer types and mechanisms of inheritance was less accurate; half (52%) of the mutation carriers marked the risk of inheriting a MMR mutation as 25% (question 8, correct answer being 50%) and two thirds (63%) recognized that female family members are at increased risk of ovarian cancer (question 5). Young age (<57 years), female sex and shorter time since genetic counseling significantly correlated with better results in univariate and multivariate analysis (Table 1). The youngest age group answered 84% of the questions correctly, compared to 71–72% among individuals >57 years (p = 0.02; three group comparison). Females answered 81% of the questions correctly, compared to 71% in males (p = 0.01). Also, time since genetic counseling was significantly associated with the results, with better knowledge among individuals who underwent genetic counseling within the last 6 years (p = 0.02; three group comparison). Sex, age and time since genetic counseling explain 32% of the variability of correct answers (R^2 ^from multivariate analysis). Education, previous cancer diagnosis and status as index individual did not significantly influence the results.

**Table 1:** Characteristics of mutation carriers in relation to correct answers

Characteristics	Number of individuals	Correct answers	Kruskal-Wallis Test	Multivariate analysis
	n = 67	Total (%)	p-value	p-value*

Overall	67	562 (76%)		
Age (22–81)			**0.02**	**0.001**
≤ 45 years	25	230 (84%)		
46–56 years	21	167 (72%)		
≥ 57 years	21	165 (71%)		
Sex			**0.01**	**0.002**
Female	38	337 (81%)		
Male	29	225 (71%)		
Education			0.51	
Elementary school	17	133 (71%)		
High School	21	177 (77%)		
University	18	160 (81%)		
Other/no data	11	92 (76%)		
Index person			0.29	
Yes	19	166 (79%)		
No	48	396 (75%)		
Time since genetic counseling (0–11)			0.08	**0.02**
≤ 3 years	26	231 (81%)		
4–6 years	23	192 (76%)		
≥ 7 years	18	139 (70%)		
Previous cancer			0.31	
Yes	28	230 (75%)		
No	39	332 (77%)		

*Linear regression model with answers as the dependent variable and age, time since genetic counseling and sex as independent variables

### Key statements among physicians

The median number of correctly answered questions among the physicians was 9 (5–11) (Additional file 1). Questions about cancer risk, colonoscopies, and genetic testing (questions 3, 6, 9 and 10) were correctly answered by 90–100% and for most of the statements the results were similar between family members and physicians. Compared to family members, physicians significantly more often gave correct answers to questions 6, 7 and 9 (which related to colonoscopies and tumor tissue for diagnostic purposes), but scored significantly worse than the family members on question 8: *individuals with HNPCC will pass the mutated gene on to 25% (1 in 4) of their children*. Only 30% of the physicians indicated that this was wrong. Regarding the risk of endometrial cancer, 77% indicated a risk for endometrial cancer and 61% indicated a risk for ovarian cancer. There were no significant differences in the outcome in relation to specialization (i.e. surgery, gynecology or oncology), employment at a university hospital or a regional hospital, sex or age.

### Risk and surveillance strategies among physicians

**Figure 1:**
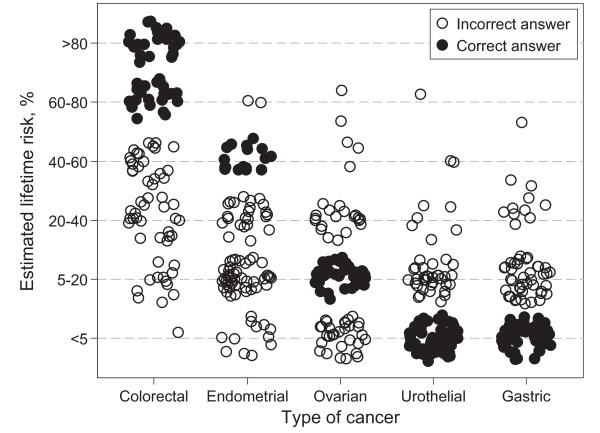
**Scatter plot of physicians estimation of the lifetime risk of various HNPCC-associated cancer types**. The physicians generally underestimated the risk of the common types of HNPCC-associated cancers; with 56% underestimating the risk of colorectal cancer and 77% of endometrial cancer. Ovarian cancer was underestimated by 29% and overestimated by 25%. The risks of rare tumor types were, on the other hand, generally overestimated; by 41% for upper tract urothelial cancer and 47% for gastric cancer. For visual purpose, independent random errors, drawn from a uniform distribution on a circle with centre (0,0), have been added to the responses.

The questions on cancer risk and surveillance posed to the physicians, demonstrated suboptimal knowledge, but did not correlate with characteristics such as age, sex and time in practise. The cumulative cancer risks were correctly indicated by less than half of the physicians; 45% for colorectal cancer, 18% for endometrial cancer, 43% for ovarian cancer, 39% for gastric cancer and 54% for upper tract urothelial cancer (Figure 1). Only 53% correctly indicated the initiation age for colonoscopies and 38% for gynecological cancer. Though surveillance for gastric cancer and upper tract urothelial cancer remains controversial, current guidelines suggest initiation at age 30–35 in affected families, whereas the majority indicated a starting age >45 years, which is higher than recommended in any guideline.

## Discussion

After the identification of disease predisposing MMR gene mutations in the early 1990'ies, genetic testing for HNPCC was established. Clinical geneticists and genetic counselors were educated, but the translation of knowledge and implementation among physicians responsible for diagnostics and surveillance has in many instances been difficult [27-29]. An increasing number of healthy at risk individuals now undergo genetic testing and though not formally patients, these individuals are dependent on health care for early cancer prevention. Individuals in HNPCC families often have experience of cancer, if not personal, perhaps in close family members. Their experiences and the vast amount of information available, not least on the internet, make them well educated with extensive knowledge about HNPCC. They thereby provide a challenge to the responsible physician and previous studies suggest that they perceive a lack of professional knowledge when discussing risks and preventive strategies with health care personnel [30].

The key statements used to assess knowledge about HNPCC revealed surprisingly similar results for at risk individuals and physicians (Additional file 1). Physicians scored significantly better on questions related to surveillance and diagnostics (questions 6, 7 and 9). Both groups revealed weaknesses related to mechanisms of inheritance as exemplified by 70% of the physicians answering that "1 of 4 children will inherit the mutated gene" (which is wrong since this figure applies to recessive disorders and the correct answer is 50% in dominantly inherited disorders). Only 30% of the physicians and 52% of the family members correctly answered this statement, and the result was indeed significantly better in the family members. Also, 25% of family members and physicians alike answered that male inheritance is more common, which is wrong since no sex difference applies to autosomal inherited disorders. The demonstration of equal and in some instances better knowledge among members of hereditary cancer families, than among physicians responsible for the management of at risk individuals, is likely to influence trust and satisfaction. These findings are in line with indications of dissatisfaction on behalf of the patients and likely to reflect frustration among physicians. Since experience among physicians did not influence the result, education in genetic medicine probably needs to be improved during medical education.

Two thirds of physicians and family members alike failed to recognize the increased risk of ovarian cancer in HNPCC, which may be related to the more recent focus on the 10–15% risk of this tumor type in female carriers. This issue, however, reflects the need to establish ways to reach out with new information to families and responsible physicians. In order to optimize management of these families, reassurance that the individual has understood the information and distribution of printed information which can be brought home and shared with other family members seem efficient. Among the mutation carriers, older individuals and males had a significantly lower number of correct scores, which may indicate that these groups need to receive information in other ways. There was also a trend for higher scores in individuals who had undergone genetic counseling within 6 years compared to those counseled a longer time ago, which may imply a need for information update; many family members ask for possibilities to learn about recent research findings related to HNPCC.

The additional questions posed to the physicians regarding surveillance programmes demonstrated that 3 out of 4 estimated a later starting age and a longer time between the examinations than recommended [5,6]. Surveillance for colorectal cancer is based on consistent results from several well designed and well performed studies, whereas there is yet insufficient evidence to support the other surveillance programmes for gynecological cancer, upper tract urothelial cancer and gastric cancer. Knowledge about the evidence and recommendations according to current international guidelines is central for physicians responsible for diagnosis and clinical management of HNPCC. Though we cannot exclude poorer results in the health care region where the data were collected, several recent studies demonstrate insufficient knowledge about hereditary cancer among physicians [27,29,31]. A comprehensive evaluation of knowledge and management of hereditary cancer, including different regions and health care systems, is called for in order to determine strategies for improved education which may lead to refined future diagnostics and management of HNPCC family members.

## Conclusion

In summary, this study reveals weaknesses in HNPCC knowledge, particularly among physicians. By tradition, the medical perspective dominates and decides on relevant and evidence based interventions. When a majority of the physicians misinterpret hereditary mechanisms, underestimate the risk of cancer, and fail to recognize HNPCC associated tumor types the likelihood of misinformation is high. Physician behaviour may also influence patient adherence to surveillance programmes, and our findings strongly suggest that improved education in genetic medicine is needed for physicians responsible for diagnosis and management of the growing number of individuals at increased risk of cancer [31-33].

## Competing interests

The authors declare that they have no competing interests.

## Authors' contributions

CC and MN conceived the idea of this study. CC, MN and KD constructed the model. KD and PB analyzed the data and interpreted the results. KD drafted the manuscript. All authors revised the paper and approved the final version. All authors take public responsibility of this paper.

## Pre-publication history

The pre-publication history for this paper can be accessed here:



## Supplementary Material

Additional file 1 **Table.** Questionnaire statements and number (%) of correct answers. Click here for file
